# Krüppel-like Factor 9 (KLF9) Suppresses Hepatocellular Carcinoma (HCC)-Promoting Oxidative Stress and Inflammation in Mice Fed High-Fat Diet

**DOI:** 10.3390/cancers14071737

**Published:** 2022-03-29

**Authors:** Adam R. Brown, Iad Alhallak, Rosalia C. M. Simmen, Stepan B. Melnyk, Melissa E. Heard-Lipsmeyer, Maria Theresa E. Montales, Daniel Habenicht, Trang T. Van, Frank A. Simmen

**Affiliations:** 1 Department of Physiology & Cell Biology, University of Arkansas for Medical Sciences, Little Rock, AR 72205, USA; adam_brown@bio-rad.com (A.R.B.); ialhallak@uams.edu (I.A.); simmenrosalia@uams.edu (R.C.M.S.); mlipsmeyer@ulm.vcom.edu (M.E.H.-L.); mariatheresa.montales@commonspirit.org (M.T.E.M.); daniel.habenicht01@utrgv.edu (D.H.); huyen.van@hcahealthcare.com (T.T.V.); 2 The Winthrop P. Rockefeller Cancer Institute, University of Arkansas for Medical Sciences, Little Rock, AR 72205, USA; 3 Arkansas Children’s Research Institute, Little Rock, AR 72202, USA; melnyksb@archildrens.org

**Keywords:** Krüppel-like factor 9, KLF9, liver, oxidative stress, pro-inflammatory, IL6, hepatocellular carcinoma, glutathione, 3-nitrotyrosine, 3-chlorotyrosine, HNE, NOX-4

## Abstract

**Simple Summary:**

The nuclear protein Krüppel-like factor 9 (KLF9) suppresses development of cancers in multiple tissues including the liver. However, a mechanistic basis for how KLF9 decreases cancer initiation/development has not been elucidated. Here, we used a mouse model in which the *Klf9* gene was ablated and placed wild-type and mutant animals of both sexes on a high-fat, obesogenic diet. We then examined how the presence or absence of KLF9 affected adiposity, as well as indices of liver oxidative stress and inflammation, and systemic oxidative stress. Results demonstrate that KLF9 is a suppressor of liver oxidative stress and inflammation which may underlie its tumor suppressive actions in liver and other tissues.

**Abstract:**

Obesity, oxidative stress, and inflammation are risk factors for hepatocellular carcinoma (HCC). We examined, in mice, the effects of Krüppel-like factor 9 (KLF9) knockout on: adiposity, hepatic and systemic oxidative stress, and hepatic expression of pro-inflammatory and NOX/DUOX family genes, in a high-fat diet (HFD) context. Male and female *Klf9^+/+^* (wild type, WT) and *Klf9*^−/−^ (knockout, KO) mice were fed HFD (beginning at age 35 days) for 12 weeks, after which liver and adipose tissues were obtained, and serum adiponectin and leptin levels, liver fat content, and markers of oxidative stress evaluated. *Klf9*^−/−^ mice of either sex did not exhibit significant alterations in weight gain, adipocyte size, adipokine levels, or liver fat content when compared to WT counterparts. However, *Klf9*^−/−^ mice of both sexes had increased liver weight/size (hepatomegaly). This was accompanied by increased hepatic oxidative stress as indicated by decreased GSH/GSSG ratio and increased homocysteine, 3-nitrotyrosine, 3-chlorotyrosine, and 4HNE content. Decreased GSH to GSSG ratio and a trend toward increased homocysteine levels were observed in the corresponding *Klf9*^−/−^ mouse serum. Gene expression analysis showed a heightened pro-inflammatory state in livers from *Klf9*^−/−^ mice. KLF9 suppresses hepatic oxidative stress and inflammation, thus identifying potential mechanisms for KLF9 suppression of HCC and perhaps cancers of other tissues.

## 1. Introduction

Obesity is a major public health issue with global rates increasing over the last several decades [1]. Obesity is a significant risk factor for multiple cancers, including hepatocellular carcinoma (HCC) [2,3,4]. Expansion of adipose tissue depots involves both increases in adipocyte size and the maturation of new adipocytes from precursor cells; nevertheless, the mechanisms of adipocyte differentiation continue to be elaborated [5]. The three-zinc-finger transcription factor Krüppel-like factor 9 (KLF9) has previously been linked to obesity and adipocyte differentiation. In East Asian populations, single-nucleotide polymorphisms (SNPs) in the human *KLF9* gene are associated with obesity [6], however, the functional consequences of these *KLF9* SNPs are unknown. In vitro studies of 3T3-L1 cells demonstrated a role for KLF9 in adipocyte differentiation. KLF9 was shown to function as a pro-adipogenic transcription factor acting synergistically with C/EBPα to activate PPARγ2 expression, which in turn promoted adipogenesis [7]. Another in vitro study demonstrated KLF9 to activate the early phase of adipogenesis by increasing the expression of C/EBPβ [8]. However, the in vivo effects, if any, of KLF9 on adiposity/obesity are not well documented [9].

Oxidative stress involves an imbalance between reactive oxygen species (ROS) buildup and the ability of a living system to detoxify these reactive intermediates [10]. Cellular/tissue buildup of ROS can increase the risk of multiple cancers, including HCC [11,12,13]. Obese patients have been found to exhibit increased levels of free radicals as well as a lower antioxidant capacity; thus, obesity contributes to systemic oxidative stress [14]. Similarly, elevated oxidative stress has been detected in obese mice [15] and a high-fat, obesogenic diet (HFD) was shown to increase oxidative stress markers in the liver, adipose tissues, and serum of mice [16].

KLF9 performs several known functions, via regulation of its target genes, in the liver. It was first isolated from rat liver and identified as a transrepressor of the *CYP1A1* gene and an inducer of SV40 early and HIV-1 long terminal repeat gene promoters [17,18]. By contrast, KLF9 transactivates the cholesterol 7α-hydroxylase (CYP7A) gene, whose product catalyzes the primary step in the hepatic conversion of cholesterol to bile acids [19], the latter a major route of cholesterol excretion from the body. Additionally, KLF9 potentiates HNF4α transactivation of hepatic CYP2D6 during pregnancy [20]. KLF9 is induced in mouse liver upon in vivo treatment with dexamethasone or with fasting and this up-regulation leads to increased serum glucose levels via enhanced gluconeogenesis [21]. The thyroid hormone triiodothyronine (T_3_) induces transcription of KLF9 in liver to activate pathways involved in stem cell renewal and differentiation, including Notch signaling [22,23].

KLF9 is implicated in suppression of liver cancers and other malignancies. *KLF9* expression was found to be reduced in hepatocellular carcinoma (HCC) compared to surrounding normal liver tissue; moreover, KLF9 inhibited cell proliferation and mobility and induced apoptosis of HepG2 cells in vitro [24]. Similarly, KLF9 was found to activate p53, thereby inhibiting proliferation and promoting apoptosis of SK-Hep1 and HepG2 HCC cells in vitro [25]. In animal models of hepato-carcinogenesis, T_3_ treatment impeded liver cancer development, in parallel with inductions in *Klf9* gene expression [26]. Interestingly, in response to high levels of oxidative stress, KLF9 was activated by NF-E2-related transcription factor 2 (NFE2L2; NRF2) to promote cell death through the elevation of intracellular ROS levels in the lung [27]. KLF9 modulation of oxidative stress-induced cell death in the lung, combined with its putative tumor-suppressive role in the liver, suggested further functional consideration within the physiological context of hepatic and systemic oxidative stress.

Here, we examined effects of *Klf9* null mutation on murine adiposity, hepatic and systemic oxidative stress, and hepatic inflammation. We induced adiposity and oxidative stress with a high-fat diet (HFD) and studied wild-type (*Klf9^+/+^*) and *Klf9* knockout (*Klf9*^−/−^*)* mice of both sexes for body weight gain, adiposity, select serum adipokine levels, numerous liver parameters (weight, lipid content, pro-inflammatory gene expression including those of *NOX*/*DUOX* genes), and biomarkers of hepatic and circulating oxidative stress [28,29]. The data identify a role for KLF9 in reducing hepatic and systemic oxidative stress and hepatic inflammatory state, independent of overt effects on adiposity, and provide potential mechanisms for tumor suppression within the hepatobiliary system.

## 2. Materials and Methods

### 2.1. Experimental Animals and Diet

Animal experiments were conducted following protocols approved by the University of Arkansas for Medical Sciences (UAMS) Institutional Animal Care and Use Committee. Wild-type (*Klf9^+/+^*, WT) and *Klf9*^−/−^ breeders (both in C57BL/6J background) were obtained from The Jackson Laboratory (Bar Harbor, ME, USA). Animals were housed in the UAMS animal facility under a 12 h light/12 h dark cycle. The *Klf9* genotype was determined using allele-specific PCR assays [30]. WT and *Klf9*^−/−^ breeder mice were fed a standard chow diet and offspring were weaned at postnatal day (PND) 21. Male and female progeny (8–10 mice per genotype/sex group) were continued on chow diet until PND 35 when they were switched to a high-fat diet (HFD). HFD (Harlan Laboratories, Madison, WI, USA) contained 45% calories from fat (lard) with casein as the protein source (Supplementary Table S1). Mice were weighed weekly and fed HFD ad libitum for 12 weeks (until PND 119) at which time they were euthanized (in the fed state). Blood, liver, and gonadal and retroperitoneal adipose tissues were collected. Liver and adipose tissues were weighed before being flash frozen prior to storage at −80 °C.

### 2.2. Histology and Immunohistochemistry (IHC)

Retroperitoneal adipose tissue was fixed in methanol–Carnoy solution (60% methanol, 30% chloroform, 10% acetic acid) for 24 h, transferred to 70% ethanol, and paraffin-embedded for sectioning. Sections (5 µm) were mounted on slides and stained with hematoxylin and eosin (H&E). Slides representing 4 female mice of each genotype were characterized for adipocyte sizes using Aperio ImageScope (Aperio Technologies Inc., Vista, CA, USA). Individual adipocyte areas were measured and the mean determined for 150–200 cells of each specimen. Liver tissue was cryo-sectioned at 5 μm thickness, stained with Oil Red O, and counterstained using hematoxylin. Images were acquired and analyzed using Aperio ImageScope. Lipid droplet staining intensity was measured for 5 male mouse livers of each group.

Methanol–Carnoy-fixed, paraffin-embedded liver tissue sections (5 µm) from male and female WT and *Klf9*^−/−^ mice (*n* = 5 per sex/genotype) were mounted on poly(lysine)-coated slides for IHC [30]. For quantification of 4-hydroxynonenal (4HNE) and 3-nitrotyrosine (3NT), sections were treated with 3% hydrogen peroxide for 30 min at room temperature and then with Citra Plus (Biogenex, Fremont, CA, USA). Sections were incubated in blocking solution (VectaStain ABC kits, Vector Laboratories, Burlingame, CA, USA) for 30 min prior to incubation with primary antibody in a humidity chamber for 24 h at 4 °C. Antibodies were: rabbit anti-4-hydroxynonenal (Abcam, Cambridge, MA, USA; 1:200) and rabbit anti-nitrotyrosine antibody (EMD Millipore, St. Louis, MO, USA; 1:300). Incubation with biotinylated anti-rabbit secondary antibody (1:200 dilution, VectaStain ABC kits, Vector Laboratories) was performed for 30 min at room temperature. Sections were stained with 3,3-diaminobenzidine tetra-hydrochloride (DAB Chromogen, Dako, Carpinteria, CA, USA) and counterstained with hematoxylin. Staining intensity was scored by color deconvolution (ImageScope, version 12.1, Leica Biosystems, Wetzlar, Germany) to separate brown (3,3-diaminobenzidine–positive stain) and blue (hematoxylin) pigments. Ten uniform fields per slide/mouse were analyzed.

For NOX4 IHC, sections were boiled in Citra Plus in Coplin jars in a microwave for a duration of 1.5 min at a high power setting and then for 10 min at low power. After cooling for 20 min at room temperature, sections were treated with 3% hydrogen peroxide, followed by incubation in UltraCruz^®^ Blocking Reagent (Santa Cruz Biotechnology, Dallas, TX, USA) for 30 min. Sections were then incubated overnight with rabbit anti-NOX4 antibody (1∶400 dilution, Abcam, Cambridge, MA, USA) at 4 °C followed by secondary anti-rabbit antibody (Vectastain Elite ABC kit, Vector Laboratories, Burlingame, CA, USA) for 30 min. Sections were stained with DAB Chromogen and counterstained with hematoxylin and slides were imaged with an EVOS^®^ FL Auto Imaging System (Life Technologies, Carlsbad, CA, USA).

### 2.3. Serum Leptin and Adiponectin

Serum leptin and adiponectin levels were determined (7–9 mice/genotype/sex) using the Mouse Leptin ELISA and the Mouse Adiponectin ELISA (Millipore Corp., Billerica, MA, USA). Individual mouse serum samples were assayed in duplicate in each assay.

### 2.4. Triglyceride Assay

Hepatic and serum triglyceride contents were measured for female mice (*n* = 7–9 mice/genotype) using the Triglyceride Colorimetric Assay Kit (Cayman Chemical, Ann Arbor, MI, USA). One hundred micrograms of liver was homogenized in 500 µL of the manufacturer’s standard diluent with 1 mM EDTA and the suspension was centrifuged. Supernatant was removed and diluted 1:5 in provided diluent solution. Ten microliters serum or 10 µL diluted liver homogenate was assayed in duplicate for each mouse.

### 2.5. Glycogen and Cholesterol Assays

Liver glycogen content of female mice was measured using the Glycogen Colorimetric Assay (BioVision, Milpitas, CA, USA). Liver homogenates (described above) were diluted 1:10 in the manufacturer’s hydrolysis buffer and 50 µL was assayed in singlet for each mouse (*n* = 7–9 mice/genotype). Hepatic and serum cholesterol levels were assayed in female mice using the Cholesterol/Cholesteryl Ester Quantitation Kit (BioVision, Milpitas, CA, USA). Samples were 3 µL serum and 50 µL liver homogenate per mouse (*n* = 7–9 mice/genotype).

### 2.6. Oxidative Stress

Serum and liver homogenates from female animals (*n* = 6 mice/genotype) were analyzed for concentrations of amino-thiols (methionine, homocysteine, cysteine, cystine, and oxidized and reduced glutathione) and 3-nitrotyrosine and 3-chlorotyrosine, all as measures of redox status. All analytes were measured by high-performance liquid chromatography (HPLC) with electrochemical detection (HPLC-ED) [31]. In brief, 100 μL of 10% meta-phosphoric acid was added to 100 μL of plasma or 100 μL (20 mg) of liver homogenate and the solution was kept on ice for 30 min. After centrifugation for 15 min at 18,000 *g* at 4 °C, supernatants were passed through a 0.2 μm nylon membrane filter and 20 μL of each was injected into the HPLC system. Analyses were performed using a HPLC-ED model 5200A and C_18_ MCM column 170 (5 μm; 4.6 × 150 mm). Metabolites were quantified using HPLC-ED software.

### 2.7. RNA Isolation and Quantitative RT-PCR (qPCR)

Total RNA was extracted from individual mouse liver and fat samples by use of TRIzol (ThermoFisher, Waltham, MA, USA). cDNA was synthesized from 1 μg of total RNA using an iScript cDNA Synthesis Kit (Bio-Rad Laboratories, Hercules, CA, USA); qPCR used iTaq Universal SYBR Green Supermix (Bio-Rad Laboratories, Hercules, CA, USA), and the Bio-Rad CFX96 Real Time System module and C1000 Touch thermal cycler. Primer sequences are listed in Supplementary Table S2. Target mRNA abundance was normalized to a factor derived from the geometric mean of expression values for 18S ribosomal RNA (*Rn18s*), β-actin (*Actb*), and TATA box binding protein (*Tbp*), calculated using the GeNorm program [32].

### 2.8. Statistical Analysis

Data (mean ± SD) were compared between WT and *Klf9*^−/−^ groups (within sex) by Student’s paired *t*-test using SigmaStat version 3.5 software (SPSS Inc., Chicago, IL, USA). Differences in the figures are indicated as * *p* ≤ 0.05, ** *p* ≤ 0.001, and # 0.1 < *p* < 0.05.

## 3. Results

### 3.1. KO of Klf9 Had No Effect on Weight Gain, Fat Deposition, or Adipocyte Size in Mice Fed HFD

**Figure 1:**
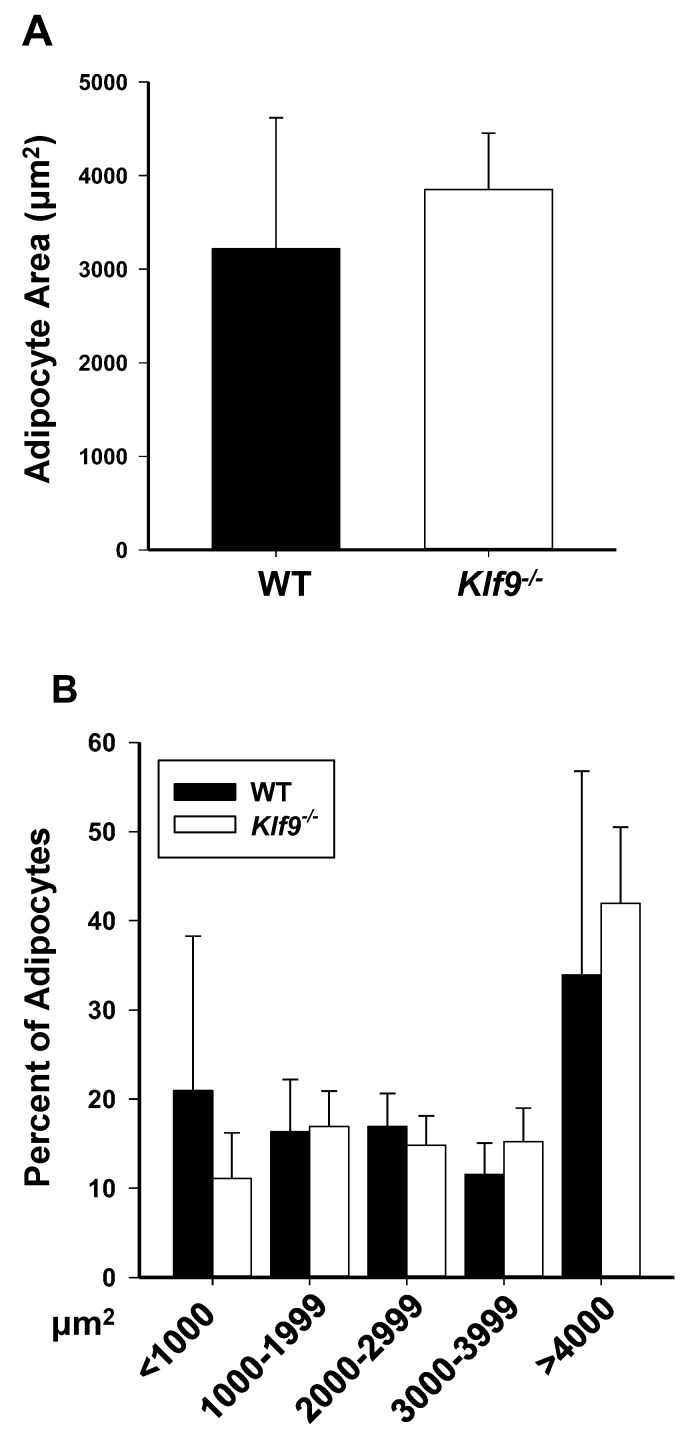
Adipocyte size distributions for retroperitoneal fat of WT and *Klf9*^−/−^ female mice are similar. (**A**) Average adipocyte size in female mouse retroperitoneal fat pads. (**B**) Adipocyte size distribution in female mouse retroperitoneal fat pads. Bar graphs are mean ± SD; *n* = 4 mice/genotype.

While KLF9 facilitates adipocyte differentiation in vitro, the in vivo role of KLF9 in promoting adipose tissue deposition has not been extensively examined. To evaluate this, male and female WT and *Klf9*^−/−^ mice were placed on HFD for 12 weeks and examined for weight gain, adiposity, and liver weight (Table 1). Fold change in body weight from PND35 was measured in male and female mice over the 12 weeks on HFD. Aside from one point at week 8 for females (PND 91) in which *Klf9*^−/−^ mice exhibited greater weight, no significant weight differences were observed between genotypes for males or females (Table 1). Similarly, retroperitoneal and gonadal fat pad weights at study termination were comparable between genotypes for both males and females (Table 1). Further, average adipocyte size (Figure 1A) and adipocyte size distribution (Figure 1B) in female retroperitoneal fat pads did not differ for WT vs. KO. Food consumption did not vary between genotypes within each sex (Figure S1).

### 3.2. Increased Liver Weights in HFD-fed Klf9 KO Mice

**Figure 2:**
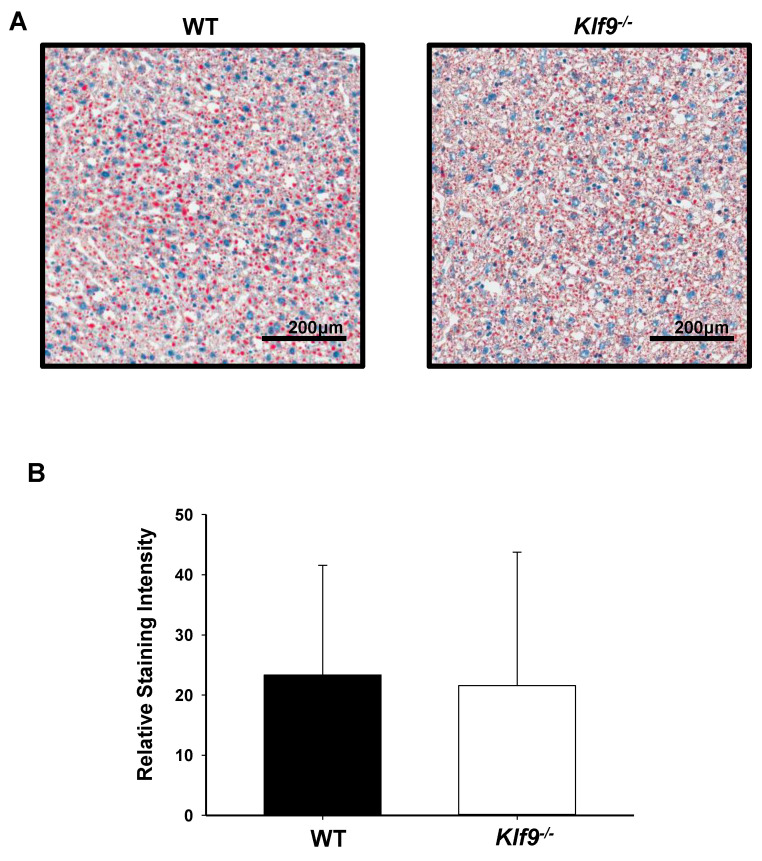
Liver lipid content, as monitored by Oil red O staining, did not differ in male WT and *Klf9*^−/−^ mice on HFD. (**A**) Representative Oil red O staining of liver of one male WT and one male *Klf9*^−/−^ mouse. (**B**) Mean staining score (*n* = 5 mice/genotype) ± SD.

**Figure 3:**
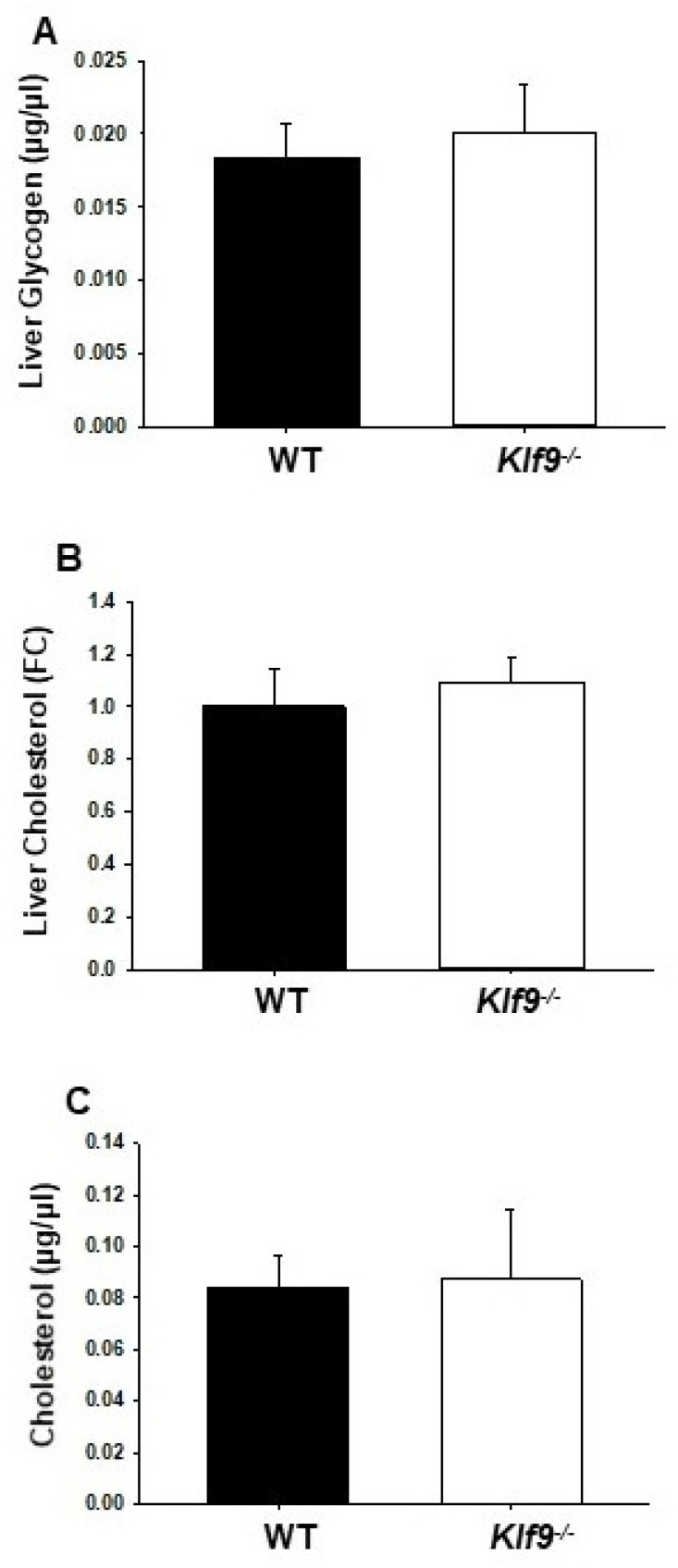
(**A**) liver glycogen and (**B**) cholesterol levels, and (**C**) serum cholesterol levels, did not differ in female WT and *Klf9*^−/−^ mice on HFD. Bar graphs represent mean ± SD (*n* = 7–9 mice/genotype).

We examined the effect of *Klf9* KO on the livers of experimental animals. Male and female *Klf9*^−/−^ mouse livers were heavier than those of corresponding WT, in both absolute weight and when normalized to body weight (Table 1). However, no change (per constant tissue weight) in liver or serum triglyceride levels was observed for female mice as a function of genotype (Table 1). Similarly, liver lipid content as a function of genotype did not differ for male mice, when evaluated by Oil red O staining (Figure 2). There were no effects of *Klf9* KO on liver glycogen, liver cholesterol, and circulating cholesterol levels of female mice (Figure 3A–C).

### 3.3. Effect of Klf9 KO on Circulating Leptin and Adiponectin Levels

**Figure 4:**
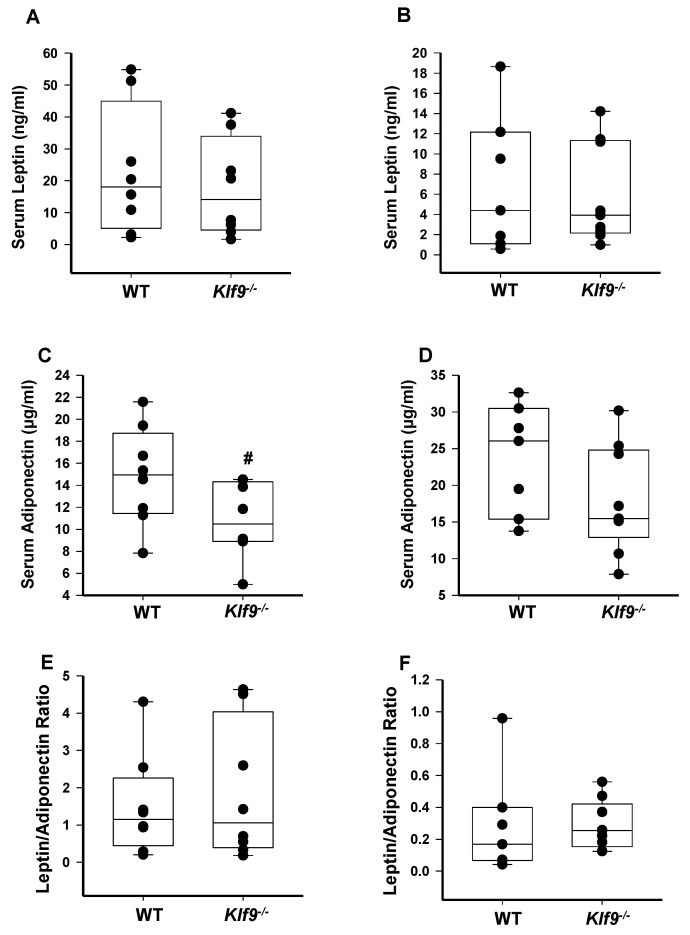
Circulating leptin and adiponectin levels in *Klf9*^−/−^ and WT mice fed HFD. Serum leptin in male (**A**) and female (**B**) WT and *Klf9*^−/−^ mice on HFD (*n* = 7–9 mice/sex/genotype). Serum adiponectin in male (**C**) and female (**D**) WT and *Klf9*^−/−^ mice on HFD (*n* = 7–9 mice/sex/genotype). Serum leptin/adiponectin ratio in male (**E**) and female (**F**) WT and *Klf9*^−/−^ mice on HFD (*n* = 7–9 mice/sex/genotype). Dots are values for the individual mice. Box plots indicate the upper and lower quartiles, interquartile range, and median (middle line); with whiskers indicating the maximum and minimum points. #, *p* = 0.065.

**Figure 5:**
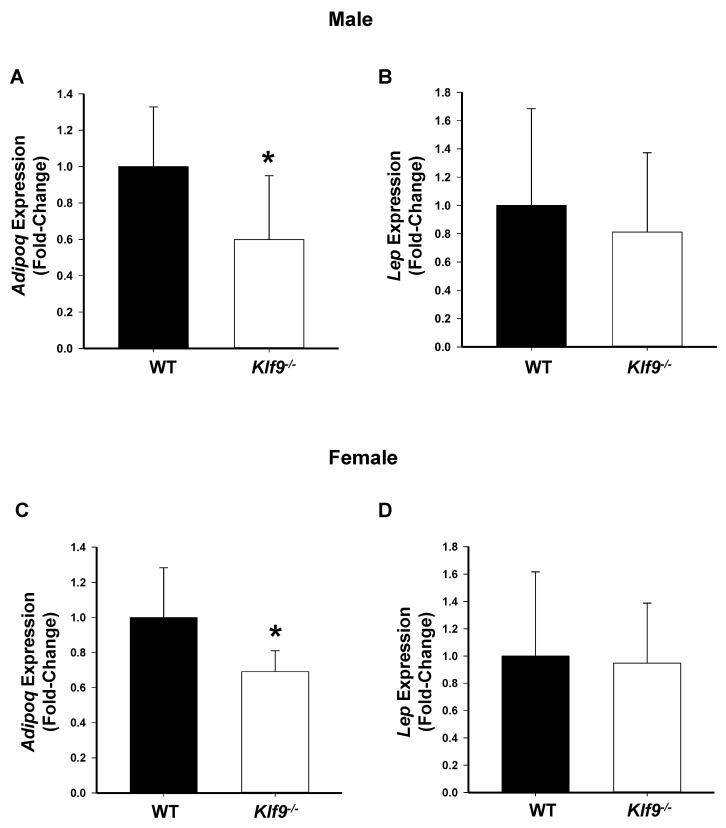
*Klf9*^−/−^ mice had decreased adiponectin mRNA abundance in gonadal fat compared to WT mice, but exhibited no difference from WT mice with respect to gonadal fat leptin mRNA abundance. (**A**,**C**) adiponectin mRNA abundance in male and female mice, respectively (*n* = 7–8/sex/genotype). (**B**,**D**) leptin mRNA abundance in male and female mice, respectively (*n* = 7–8/sex/genotype). Bar graphs are mean ± SD. * *p* ≤ 0.05, significant difference between genotypes.

Consumption of a high-fat diet can lead to increased leptin secretion by fat cells and the condition of leptin resistance, resulting in hyper-leptinemia [33]. High leptin levels, in turn, have been associated with increased cancer cell proliferation, reduced cancer cell apoptosis, and increased tumor invasion [34]. Conversely, adiponectin is negatively correlated with body fat mass, and higher circulating adiponectin levels are associated with reduced risk of several types of obesity-related malignancies [35]. We therefore evaluated the effects of KO of *Klf9* on serum leptin and adiponectin concentrations in male and female mice. No significant changes were found for serum leptin or adiponectin concentrations between genotypes within either sex, although male *Klf9*^−/−^ mice exhibited a trend (*p* = 0.064) toward reduced circulating adiponectin concentration relative to WT (Figure 4A–D). Genotype differences for systemic leptin/adiponectin ratio of either sex were also not observed (Figure 4E,F). Interestingly, adiponectin mRNA abundance was significantly lower in gonadal fat of male and female *Klf9*^−/−^ mice compared to WT counterparts (Figure 5A,C). In contrast, leptin mRNA abundance was unaffected by genotype for both sexes (Figure 5B,D).

### 3.4. Increased Oxidative and Nitrosative Stress in Livers of Klf9 KO Mice

**Figure 6:**
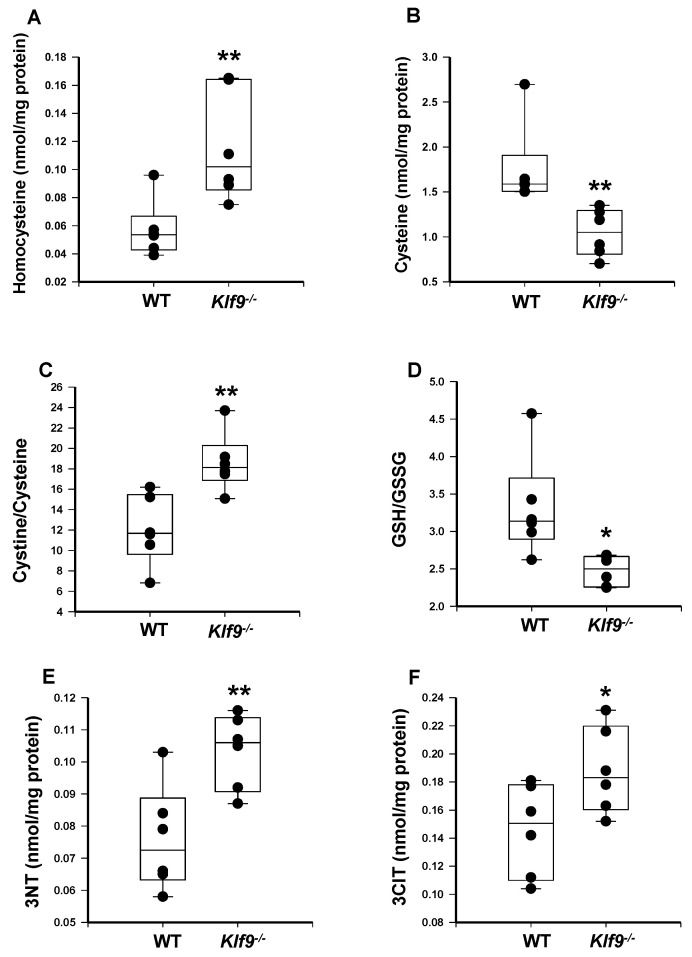
HFD-fed *Klf9*^−/−^ female mice exhibit increased hepatic oxidative and nitrosative stress compared to similarly fed WT female mice. (**A**) homocysteine, (**B**) cysteine, (**C**) cystine/cysteine, (**D**) GSH/GSSG, (**E**) 3-nitrotyrosine (3NT), and (**F**) 3-chlorotyrosine (3CIT) in liver (*n* = 6 female mice/genotype) were measured by HPLC coupled with coulometric electrochemical detection. Dots are values for the individual mice. Box plots indicate the upper and lower quartiles, interquartile range, and median (middle line); with whiskers indicating the maximum and minimum points. * *p* ≤ 0.05, ** *p* ≤ 0.001.

**Figure 7:**
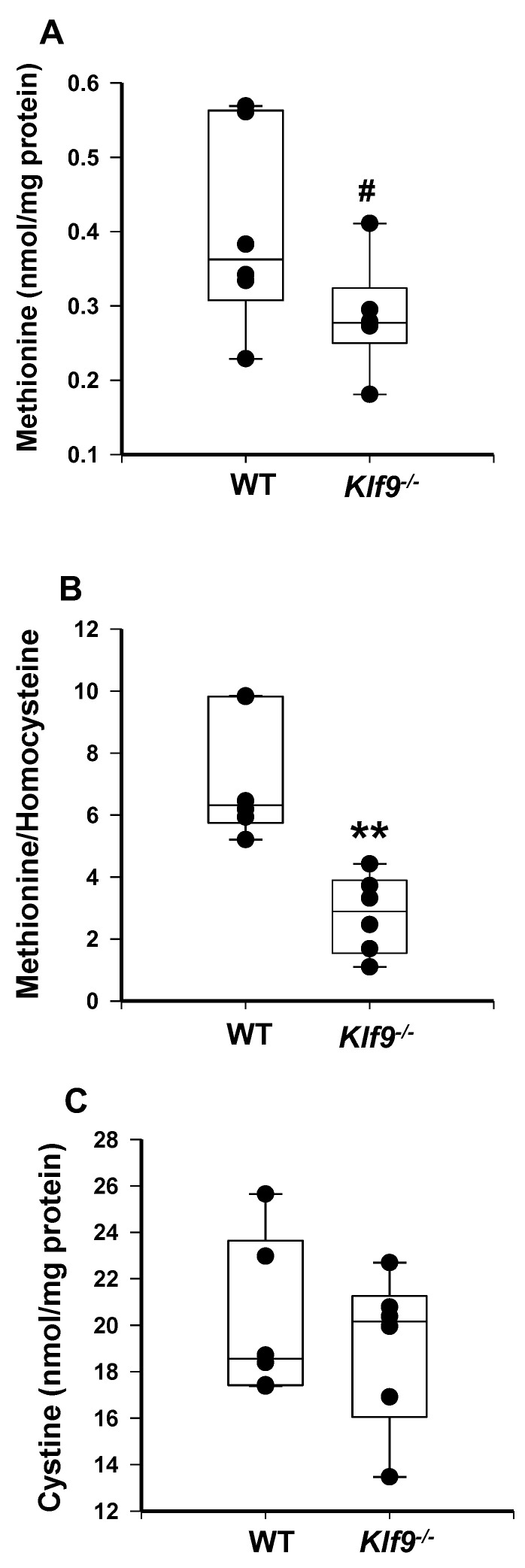
Female *Klf9*^−/−^ mice have reduced liver methionine/homocysteine ratio compared to WT mice, when fed HFD. (**A**) Methionine, (**B**) methionine/homocysteine ratio, and (**C**) cystine in female WT and *Klf9*^−/−^ mouse livers were measured by HPLC coupled with coulometric electrochemical detection. Dots represent values for individual mice (*n* = 6/genotype). Values are indicated as ** *p* ≤ 0.001 and # 0.1 < *p* < 0.05.

We next examined markers of oxidative stress, nitrosative stress, and inflammation-induced protein damage in female WT and *Klf9*^−/−^ mouse livers. The initial assay was by HPLC coupled with coulometric electrochemical detection. Liver homocysteine levels were elevated, while methionine levels showed a trend for reduction (*p* = 0.092) with *Klf9* KO (Figure 6A and Figure 7A). This translated to a highly significant reduction in liver methionine/homocysteine ratio in *Klf9*^−/−^ mice (Figure 7B). *Klf9*^−/−^ mouse livers exhibited a significant reduction in free cysteine, with no change in cystine level (Figure 6B and Figure 7C), resulting in a highly significant increase in the cystine/free cysteine ratio (Figure 6C). Further, the hepatic ratio of reduced glutathione to oxidized glutathione was significantly decreased in *Klf9*^−/−^ mouse liver (Figure 6D). Conversely, levels of 3-nitrotyrosine (3NT) and 3-chlorotyrosine (3ClT) were significantly elevated in *Klf9*^−/−^ mouse livers (Figure 6E,F).

**Figure 8:**
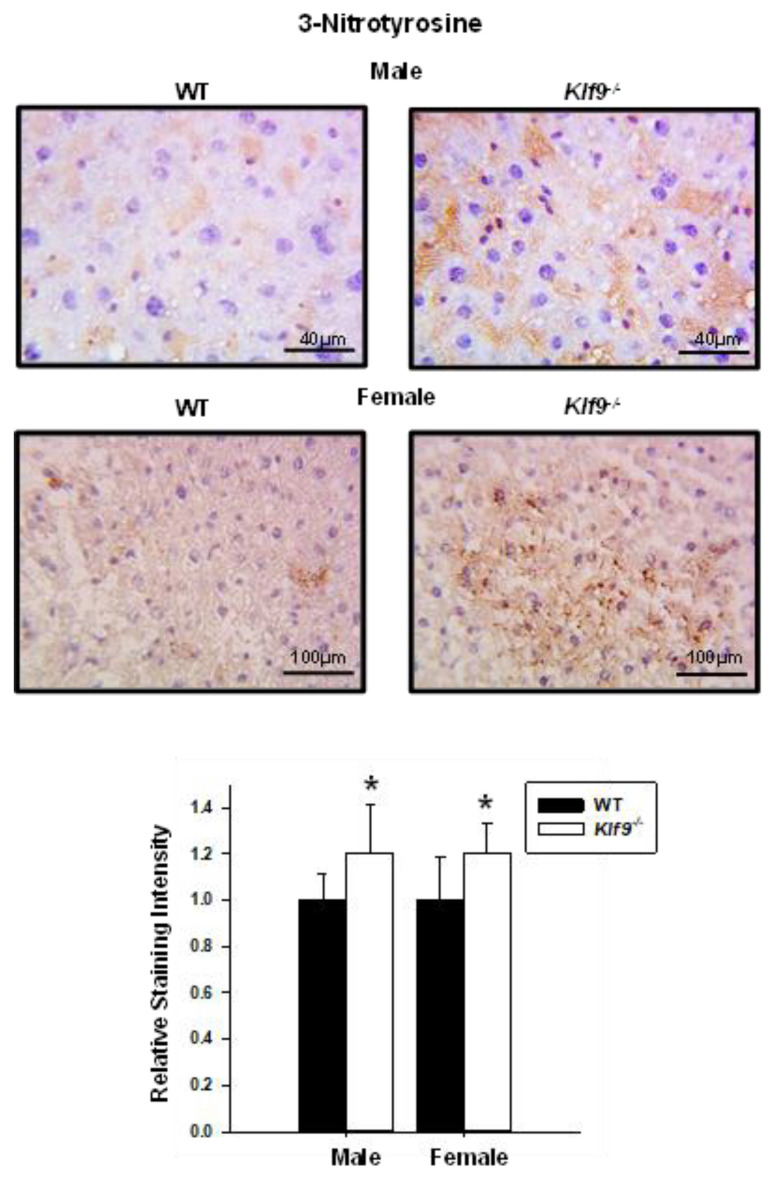
Increased nitrosative stress in livers of HFD-fed male and female *Klf9*^−/−^ mice. IHC of 3-nitrotyrosine (3NT) in livers of HFD-fed male and female WT and *Klf9*^−/−^ mice (*n* = 4–5/sex/genotype) was performed (one representative section of an animal of each sex and genotype is shown in upper panels). Two sections for each mouse underwent IHC and 5 uniform fields per section were quantitatively analyzed using ImageScope (Materials and Methods). Bar graphs represent mean staining score ± SD. * *p* ≤ 0.05.

**Figure 9:**
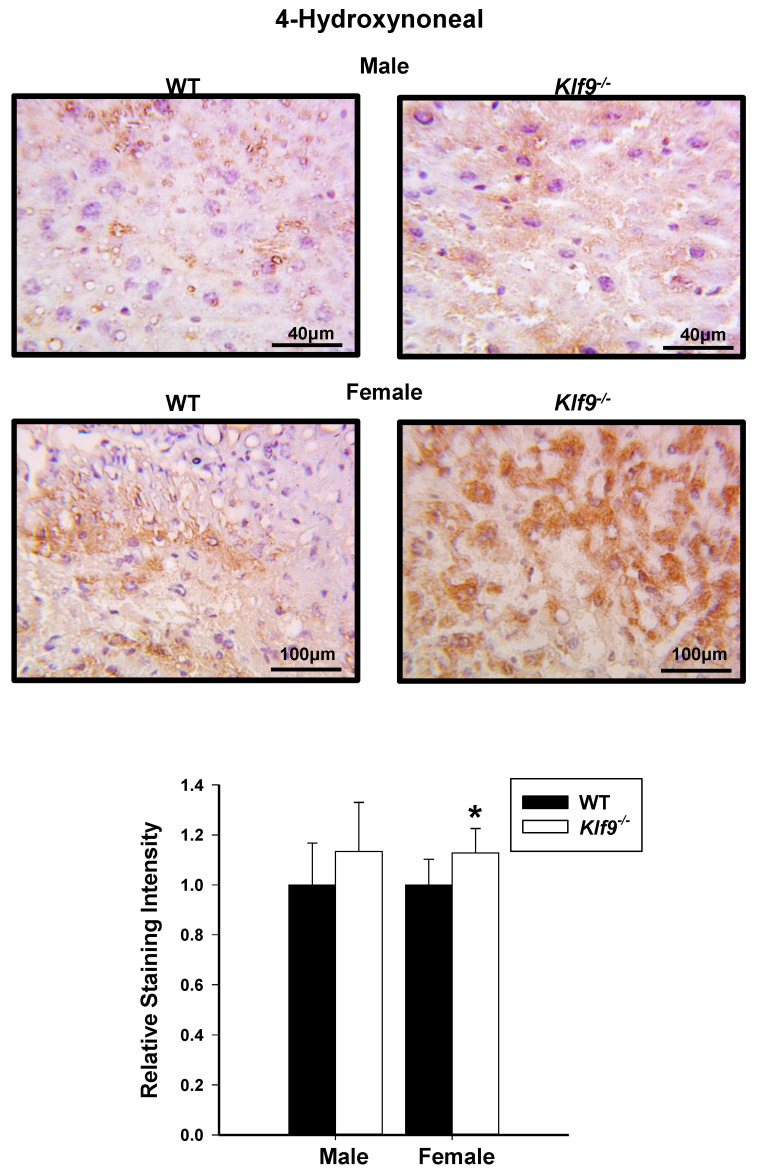
IHC for lipid peroxidation in livers of HFD-fed male and female *Klf9*^−/−^ mice. IHC of 4-hydroxynonenal (4HNE) in livers of HFD-fed male and female WT and *Klf9*^−/−^ mice (*n* = 4–5/sex/genotype) was performed (one representative section of an animal of each sex and genotype is shown in upper panels). Two sections for each mouse liver underwent IHC and 5 uniform fields per section were quantitatively analyzed using ImageScope software. Bar graphs represent mean staining score ± SD. * *p* ≤ 0.05.

To confirm the increased nitrosative stress and to also examine lipid peroxidation status, liver tissue sections (WT, *Klf9*^−/−^, males, females) were subjected to IHC for 3NT and 4-hydroxynonenal (4HNE). Correspondent with the HPLC data, increased 3NT immunoreactivity was observed for both female and male *Klf9*^−/−^ livers relative to WT (Figure 8). Immunostaining for 4HNE was significantly increased in the livers of female *Klf9*^−/−^ mice compared to WT (Figure 9). However, 4HNE levels did not differ in male mouse livers of the two genotypes (*p* = 0.14) (Figure 9).

**Figure 10:**
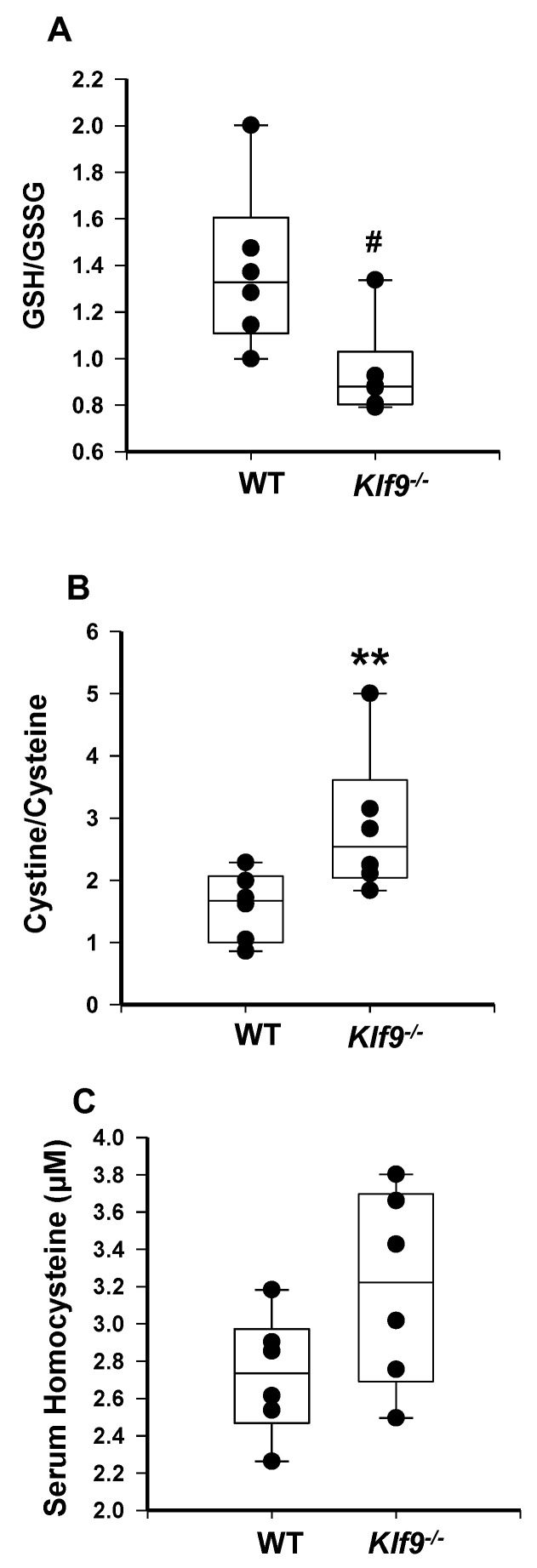
*Klf9*^−/−^ female mice have increased serum oxidative stress compared to WT female mice. Serum ratios of GSH/GSSG (**A**) and cystine/cysteine (**B**), and serum levels of homocysteine (**C**) were measured in female WT and *Klf9*^−/−^ mouse serum by HPLC coupled with coulometric electrochemical detection. Dots represent values for individual mice (*n* = 6/genotype). Box plots indicate the upper and lower quartiles, interquartile range, and median (middle line); with whiskers indicating the maximum and minimum points. ** *p* ≤ 0.001 and # 0.1 < *p* < 0.05.

**Figure 11:**
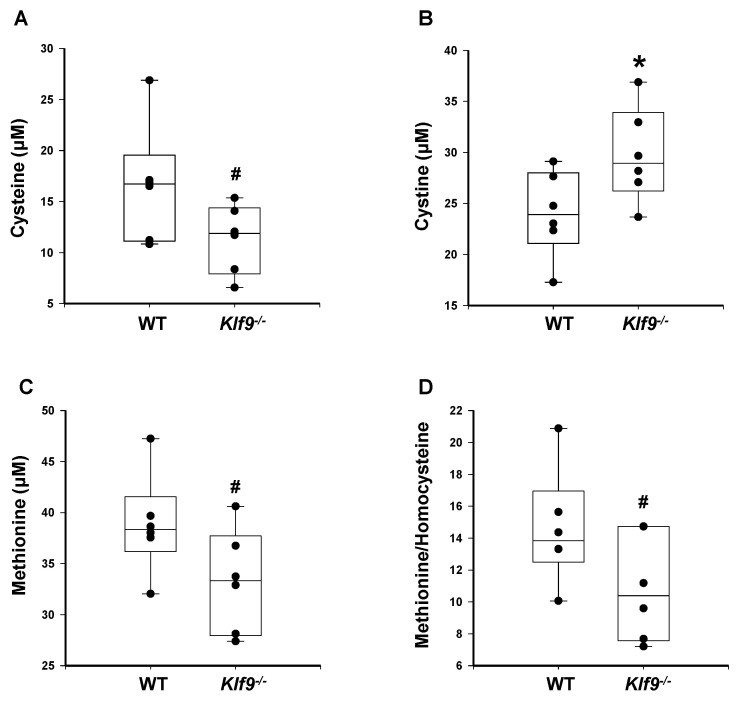
HFD-fed female mice null for *Klf9* exhibit trends toward reduced serum cysteine, reduced serum methionine, and reduced methionine/homocysteine ratio; as well as significantly increased serum cysteine, when compared to WT. Serum levels of (**A**) cysteine, (**B**) cystine, (**C**) methionine, and (**D**) methionine/homocysteine (ratio) in female WT and *Klf9*^−/−^ mouse livers were measured by HPLC coupled with coulometric electrochemical detection. Dots represent values for individual mice (*n* = 6/genotype). Box plots indicate the upper and lower quartiles, interquartile range, and median (middle line); with whiskers indicating the maximum and minimum points. Differences are indicated as * *p* ≤ 0.05 and # 0.1 < *p* < 0.05.

Next, we examined if alterations in select biomarkers of serum oxidative stress accompanied those noted for the liver (and potentially a result of blood perfusing the liver). Ratios of reduced glutathione to oxidized glutathione tended to decrease in female *Klf9*^−/−^ mouse serum (Figure 10A). Similarly, *Klf9* KO mice showed a trend (0.05 < *p* < 0.10) toward reduced free cysteine in the serum (Figure 11A), while serum cystine levels were significantly elevated (Figure 11B). Hence, serum cystine/cysteine ratios were elevated in *Klf9* KO mouse serum (Figure 10B) as noted for livers. Homocysteine levels were numerically (but non-significantly) increased (*p* > 0.10) (Figure 10C), while methionine levels and methionine/homocysteine ratios tended to decrease for *Klf9* KO serum (Figure 11C,D).

### 3.5. Hepatic Genes

**Figure 12:**
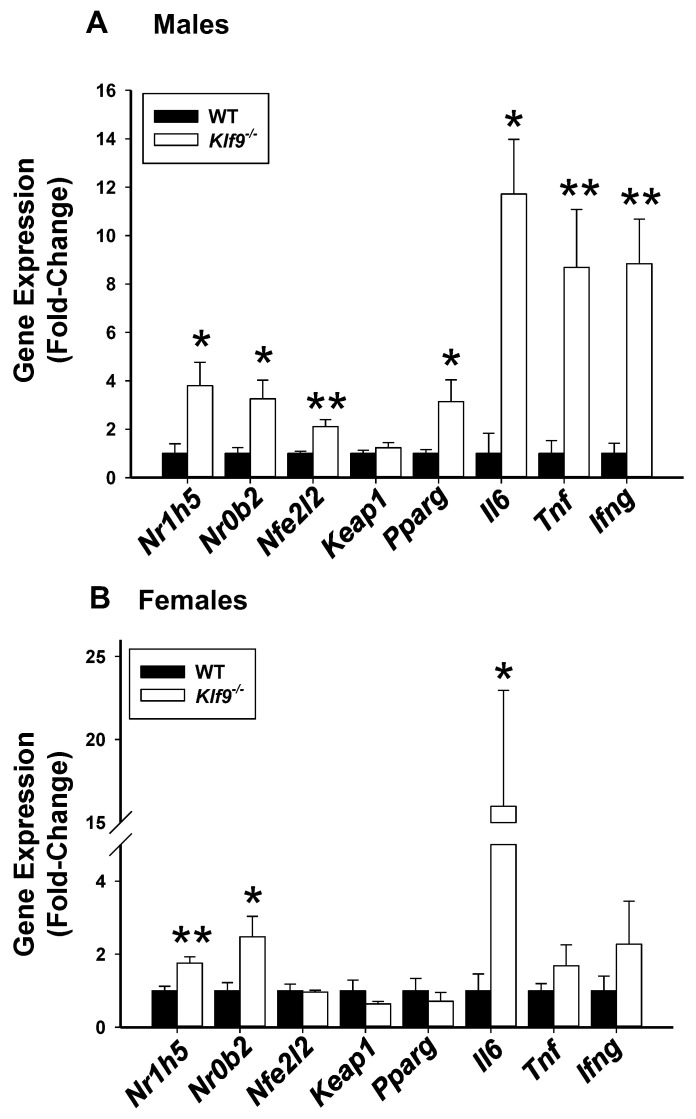
Increased mRNA abundance, in livers, of *Nr1h5* (FXRβ), *Nr0b2* (SHP) and *Il6* (Interleukin-6) genes is common to HFD-fed male and female *Klf9*^−/−^ mice; whereas increased mRNA abundance of *Nfe2l2* (NRF2), *Pparg* (PPARγ), *Tnf* (TNFα) and *Ifng* (IFNγ) is characteristic for livers of male *Klf9*^−/−^ mice, compared to WT counterparts. Bar graphs represent abundance relative to WT animals (mean ± SD; females, *n* = 8–9/genotype; males, *n* = 5–6/genotype). * *p* ≤ 0.05, ** *p* ≤ 0.001.

We quantified hepatic mRNA levels for several nuclear receptor genes (*Nr1h5*, *Nr0b2*, *Pparg*), antioxidant pathway regulator genes (*Nfe2l2*, *Keap1*), and pro-inflammatory genes (*Il6*, *Tnf*, and *Ifng*), all of which have been implicated in liver cyto-protection, regeneration, and growth. Male *Klf9*^−/−^ mouse livers exhibited increased steady-state levels of all studied genes with the exception of *Keap1*, when compared to WT counterparts (Figure 12A). Female mice null for *Klf9* had increased steady-state levels of *Nr1h5*, *Nr0b2*, and *Il6* when compared to WT (Figure 12B). These data suggest a more heightened pro-inflammatory state in male than female *Klf9*^−/−^ mouse livers under the HFD paradigm, when compared to WT counterparts.

**Figure 13:**
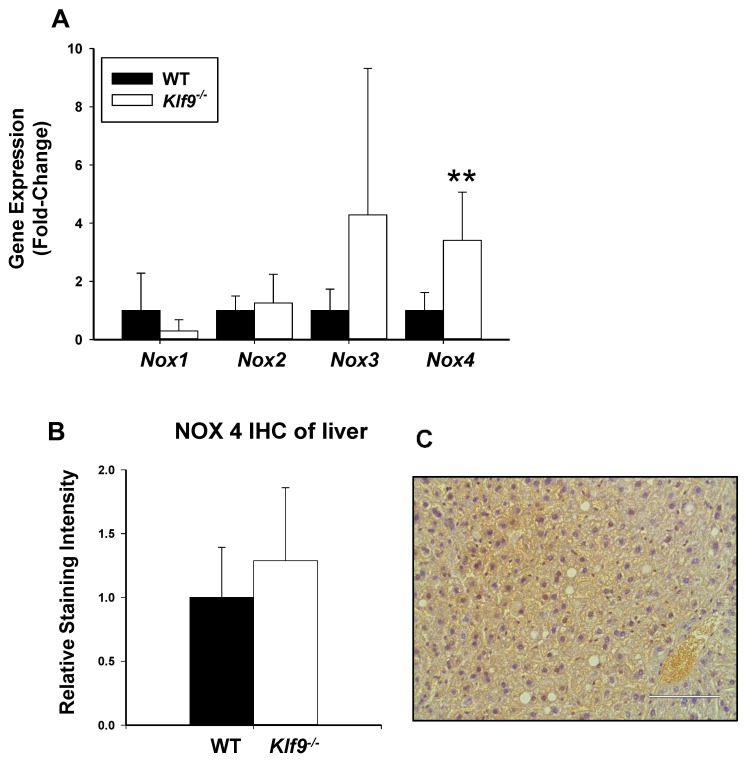
Increased *Nox4* mRNA, but not NOX4 protein abundance, in livers of HFD-fed, male *Klf9*^−/−^ mice. (**A**) Bar graphs represent mRNA level relative to WT animals (mean ± SD; *n* = 5–6 mice/genotype; ** *p* ≤ 0.001. (**B**) IHC of liver NOX4; no significant difference between WT and *Klf9*^−/−^ mice (*n* = 5/group). (**C**) Representative NOX4 IHC image (WT male; scale bar is 100 µm).

Lastly, we examined hepatic mRNA abundance for four members (NOX1, NOX2, NOX3, and NOX4) of the NOX/DUOX family of proteins, since these are expressed in the liver where they contribute to hepatic ROS generation [28,29]. *Nox4* mRNA was more highly expressed in *Klf9*^−/−^ than WT livers (Figure 13A); by contrast, no significant differences in *Nox1*, *Nox2*, and *Nox3* mRNA abundance were observed with genotype. However, unlike the differing pattern of *Nox4* mRNA abundance with genotype, IHC staining intensity for the corresponding protein was comparable between *Klf9*^−/−^ and WT livers (Figure 13B,C).

## 4. Discussion

KLF9 is an evolutionarily well-conserved transcriptional regulator implicated as a tumor/metastasis suppressor in liver [24,25], endometrium [36,37], colon and rectum [38], breast [39], glioblastoma [40], ovary [41], and osteosarcoma [42]. Nevertheless, the underlying mechanism of its tumor-suppressive action for any given tissue remains undefined. The HFD-induced obesity model in C57BL/6J mice is an important tool for understanding the pathology of obesity and how this impacts cancer risk, as these mice develop obesity, hyperglycemia, and hyperinsulinemia when fed HFD ad libitum [43]. We used this model to evaluate the in vivo effects of *Klf9* KO on obesity/adiposity and related parameters including hepatic lipid content, circulating adipokine concentrations, and hepatic and systemic oxidative stress, all of which are considered to positively influence HCC risk. Given established significant differences in hepatic metabolism and gene expression between sexes of both humans and mice [44,45], we conducted the study in mice of both genders. Our results provide support to the emerging concept of KLF9 as an important regulator of local and systemic oxidative stress and ROS-induced cell responses including inflammation [27,46], and which may underlie its tumor-suppressive effects in liver and other tissues.

A surprising finding was the lack of effect of global *Klf9* KO on weight gain, fat deposition, adipocyte size, and adipocyte size distribution in mice of either sex. The absence of changes in hepatic fat content and in circulating triglyceride levels is consistent with these findings, since hepatosteatosis and hypertriglyceridemia are associated with obesity [47,48]. Moreover, serum levels of leptin and adiponectin, which are, respectively, proportional and inversely proportional to body fat mass [49,50], were comparable between WT and *Klf9* KO mice.

The discrepancy between the reported modulatory role for KLF9 in adipocyte development in vitro (Introduction), and the lack of alteration in adiposity with *Klf9* knockout in vivo as shown here, may be accounted for, in part, by compensatory mechanisms operative only in vivo. Members of the KLF family are known to exhibit compensatory functions upon loss of expression of other family members. One relevant example is that of loss of KLF13 being functionally compensated for by the highly homologous KLF9 in the peri-implantation mouse uterus [51]. Additionally, KLFs 1 and 2 have compensatory roles in controlling embryonic β-*globin* gene expression and primitive erythropoiesis in the mouse [52]. As transcription factors, KLF family members are known to modulate expression of microRNAs and to be themselves targets of microRNAs [53,54,55]. Thus, loss of *KLF9* may conceivably lead to alterations in steady state levels of microRNAs that affect expression of other genes so as to maintain adipogenesis, although this is speculative.

Non-alcoholic fatty liver disease (NAFLD) describes several related pathological states associated with excessive fat accumulation in the liver (hepatosteatosis) and in the absence of excessive alcohol consumption; NAFLD is a manifestation of obesity and metabolic syndrome and has been positively associated with risk/development of HCC [56,57]. The noted increase in liver weights of both male and female mice null for KLF9 initially suggested the possibility of greater steatosis in the livers of the *Klf9*^−/−^ mice; however, Oil Red O and H&E staining as well as triglyceride assay did not show any significant differences between genotypes. Additionally, the liver weight increase could not be explained by increased hepatic glycogen or cholesterol storage. Roles for KLF family members have been established for stem cells, organogenesis, and early development [58]. In particular, triiodothyronine (T_3_) is a potent inducer of *KLF9* in human embryonic stem cells (hESCs) and human induced pluripotent stem cells (hiPSC) during differentiation to definitive endoderm and hiPSC-derived hepatocytes [23]. KLF9 cooperates with thyroid hormone receptor (TR) to regulate Notch pathway genes in hESCs and hiPSCs [23] and KLF9 is a mediator of neural development in response to glucocorticoids [59]. KLF9 may therefore serve yet unknown functions in moderating hepatic development and final adult-stage liver size in mice, although this remains unexplored.

In an effort to understand the effect of *Klf9* KO on liver weight, we searched for parallels with the phenomenon of liver regeneration. Partial hepatectomy in rodents leads to rapid liver regeneration, a process in the mouse that is accompanied by inhibition of thyroid hormone target genes in liver including *Klf9* [22]. *Nr1h5* (FXRβ) and thyroid receptor α gene expression are markedly down-regulated during the initial proliferative phase of mouse liver regeneration [60]. Our novel observation of increased *Nr1h5* gene expression with hepatomegaly of *Klf9* KO mice of both sexes may parallel liver regeneration with partial hepatectomy. Induction of *Ifng* (IFNγ) also is associated with the proliferative phase of liver regeneration [61]. Similarly, the hepatocyte-expressed *Il6* (IL-6) gene is rapidly induced by partial hepatectomy in rats [62]. The induction of *Ifng* and *Il6* gene expression in male *Klf9* KO livers and that of the *Il6* gene in female *Klf9* KO livers may indicate a normally suppressive effect of KLF9 on liver cell proliferation, an effect that might be predicted to lead to larger livers in *Klf9* KO mice as was observed here. Our observation of a potential linkage between KLF9 and IL-6 is also noteworthy since IL-6 is a known liver mitogen implicated in liver carcinogenesis [63,64]. IL-6 may facilitate tumor metastasis via induction of glutathione release from hepatocytes and inter-organ transfer to metastatic foci [65], providing an experimentally testable connection between KLF9, IL-6, glutathione, and cancer dissemination in future studies.

A buildup of homocysteine is believed to cause auto-oxidation of thiol groups to generate hydrogen peroxide and the reactive oxygen species superoxide and hydroxyl radical, leading to cellular oxidative stress [66,67]. Reduced glutathione is considered to be the most important intracellular defense against oxidative stress. Buildup of ROS within the cell can lead to a decrease in the ratio of reduced glutathione (GSH) to oxidized glutathione (GSSG), a recognized measure of cellular antioxidant ability [68]. The observed decrease in the GSH to GSSG ratio suggests the accumulation of ROS in the livers of *Klf9*^−/−^ compared to WT mice. The increase in cystine/cysteine ratios in liver tissue and serum of *Klf9* KO mice also aligns with the increase in oxidative stress with loss of KLF9.

Buildup of ROS is often accompanied by accumulation of nitrogenous free radicals such as nitric oxide (NO) or peroxynitrite anion (ONOO^−^), known as reactive nitrogen species (RNS), which react with different types of molecules to form toxic products [69]. *Klf9*^−/−^ mice exhibited increased liver nitrosative stress compared to WT counterparts as indicated by the increased levels of 3NT, a potential biomarker of hepato-carcinogenesis [70]. Continuous exposure of cells to oxidative and nitrosative stress can lead to chronic inflammation. Increased inflammation can in turn promote additional buildup of free radicals in a vicious cycle, further contributing to cell damage [71]. In this regard, elevated levels of 3ClT, a marker for neutrophil-mediated myeloperoxidase damage, were detected in *Klf9*^−/−^ livers, reflecting increased hepatic inflammation [72,73]. Myeloperoxidase is abundantly expressed in neutrophil granulocytes and uses hydrogen peroxide (H_2_O_2_) manufactured by phagocytes to generate microbicidal oxidants and tyrosyl radicals, which contribute to lipid peroxidation. Accordingly, *Klf9*^−/−^ livers showed increased staining for 4HNE, a major aldehydic end product of lipid peroxidation and also a marker of oxidative stress [74,75]. Given the collective evidence, the increased liver weights may be ascribed, at least in part, to the proliferative and/or anti-apoptotic effects of oxidative stress. ROS activates multiple transcription factors including AP-1, NF-κB, HIF-1α, p53, PPARγ, β-catenin/Wnt, and Nrf2, which in turn increase expression of growth factors and pro-inflammatory cytokines, leading to cell proliferation and increased risk of neoplasia [13].

Elevations in circulating homocysteine coincide with NAFLD, and increase in a manner dependent on the degree of liver damage [76,77]. Indeed, circulating homocysteine can induce oxidative damage in the liver [78]. It seems likely that the trend toward increased circulating levels of homocysteine observed in *Klf9*^−/−^ mice was due to the increased hepatic oxidative stress; the liver-driven increase in systemic oxidative stress may therefore have wider implications for oxidative damage in other organs though exposure to oxidative stress via the vasculature. In this regard, the decreased GSH/GSSG ratio in *Klf9*^−/−^ serum is likely due, in part, to efflux of oxidized and reduced glutathione from the liver into the bloodstream [79].

Multiple mechanisms have been revealed for KLF family members in modulation of oxidative stress responses. Hydrogen peroxide can increase expression of *KLF4* to up-regulate *BAX* and down-regulate *BCL-2* in K562 erythroleukemia cells and peripheral blood mononuclear cells [80]. Similarly, KLF4 decreased cell viability following ROS-induced DNA damage, increased anoikis and repressed pro-survival baculoviral IAP repeat-containing 5 (*BIRC5)* in esophageal cancer cells [81]. Overexpression of KLF15 in mouse adipose tissue led to increased local oxidative stress via suppression of stearoyl-CoA desaturase 1 (SCD1) [82]. Further, KLF11 suppressed expression of the ROS scavenger genes superoxide dismutase 2 (*SOD2*) and catalase 1 in pancreatic cancers [83]. Activation of KLF9 by NF-E2-related transcription factor 2 (*Nfe2l2*) was demonstrated in response to oxidative stress, leading to elevation of intracellular ROS and cell death in the lung [27]. The latter observation aligns with the increase in *Nfe2l2* transcripts in *Klf9* KO male but not female livers, suggesting a sex-specific compensatory mechanism for anti-oxidative stress.

It is interesting that sexual dimorphism was evident for expression of some hepatic gene upon loss of the *Klf9* gene. KLF9 has not been extensively examined with regard to sexual dimorphism of liver gene expression, although it is a well-known glucocorticoid-responsive factor and circadian clock participant, as well as being a mediator of estrogen action (in concert with estrogen receptors) [30,36,37,59,84]; all of which factor into the regulation of the large sex-specific expressed gene sets in murine liver [85]. Additionally, in this regard, KLF9 is a member of an estrogen receptor α-mediated network of female regulatory genes in mouse liver [85].

## 5. Conclusions

Utilizing an HFD-fed model of WT and *Klf9* null mice, we showed that KLF9 represses hepatic and systemic oxidative stress and hepatic inflammation state, while having no overt effects on obesity/adiposity. However, we did observe lowered adiponectin gene expression in *Klf9* KO adipose tissue, which is keeping with transactivation of the adiponectin gene promoter by KLF9 [86]. Future studies should address the nature of the serum adiponectin size variants (and their circulating abundance) in mice as a function of *Klf9* status. We propose that KLF9 functions as a suppressor of tumorigenesis in the liver through two mechanisms—first, by its suppression of proliferative and anti-apoptotic mechanisms associated with ROS and, second, by its suppression of hepatic IL-6 production (and by inference of circulating IL-6). Parallel analyses of other tissues for alterations in redox state parameter(s) and IL-6 status as a function of KLF9 genotype may provide consensus pathway(s) for its anti-tumorigenic functions [38,87]. Lastly, the induction of the adiponectin gene by KLF9 is clearly of follow-up interest from the related contexts of tumor suppression and insulin sensitivity (anti-diabetes).

## Figures and Tables

**Table 1:** Phenotypic indices of WT and *Klf9* KO mice fed HFD.

**Fold change in body weight (BW) after 12 wk on HFD; *n* = 8–9 mice/sex/genotype**			
WT males	*Klf9*^−/−^ males	WT females	*Klf9*^−/−^ females
1.775 + 0.204	1.851 + 0.263	1.416 + 0.0984	1.490 + 0.0832
Males (WT plus *Klf9^−/−^*)		Females (WT plus *Klf9^−/−^*)	
1.811 + 0.228		1.455 + 0.0957 **^1^**	
** Retroperitoneal fat weight (normalized to BW); *n* = 8–9 mice/sex/genotype **			
WT males	* Klf9^−/−^* males	WT females	* Klf9^−/−^* females
1 + 0.299	0.868 + 0.400	1 + 0.732	1.237 + 0.681
** Gonadal fat weight (normalized to BW); *n* = 8–9 mice/sex/genotype **			
WT males	* Klf9^−/−^* males	WT females	* Klf9^−/−^* females
1 + 0.368	0.895 + 0.444	1 + 0.610	0.887 + 0.425
** Liver weight (normalized to BW); *n* = 8–9 mice/sex/genotype **			
WT males	* Klf9^−/−^* males	WT females	* Klf9^−/−^* females
1 + 0.134	1.194 + 0.185 **^2^**	1 + 0.115	1.209 + 0.221 **^3^**
** Liver triglycerides (mg/dL) **		** Serum triglycerides (mg/dL) **	
WT females	* Klf9^−/−^* females	WT females	* Klf9^−/−^* females
137.3 + 61.1	144.3 + 64.1	145.7 + 29.2	126.7 + 37.6

**^1^** *p* = 0.000002, **^2^** *p* = 0.03, **^3^** *p* = 0.04.

## Data Availability

The data presented in this study are available on request from the corresponding author.

## Supplementary Materials

The following supporting information can be downloaded at: https://www.mdpi.com/article/10.3390/cancers14071737/s1, Figure S1: HFD consumption of WT and *Klf9*^−/−^ mice did not differ; Table S1: Composition of HFD (g component/kg diet); Table S2: Sequences of DNA primers used in qPCR analyses.
