# Supplementary material for: Krüppel-like Factor 9 (KLF9) Suppresses Hepatocellular Carcinoma (HCC)-Promoting Oxidative Stress and Inflammation in Mice Fed High-Fat Diet
Source: Cancers (Basel). 2022 Mar 29;14(7):1737. doi: 10.3390/cancers14071737 (PMC8996893; doi:10.3390/cancers14071737)
Supplement: Supplementary file 1 [file cancers-14-01737-s001.zip › cancers-1624654-supplementary.pdf]

cancers MDPI

Supplementary Material

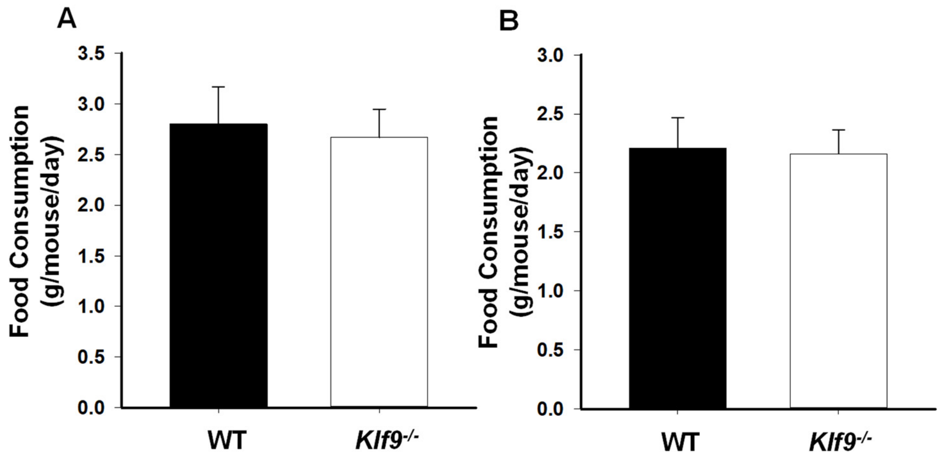

Figure S1. HFD consumption of WT and *Klf9*$^{-/-}$ mice did not differ. (A) male mice (n = 8–9/genotype); (B) female mice (n = 8–9/genotype). Bar graphs are mean ± SD.

Table S1. Composition of HFD (g component/kg diet).

Component	g/kg
Casein	210
L-cystine	3
Corn starch	160
Maltodextrin	150
Sucrose	150
Lard	210
Corn oil	20
Cellulose	28.96
Mineral Mix, AIN-93G-MX (94046)	50
Vitamin Mix, AIN-93-VX (94047)	15
Choline bitartrate	3
TBHQ, antioxidant	0.04

Table S2. Sequences of DNA primers used in qPCR analyses.

Mouse gene	Forward primer	Reverse primer
Actb	CGTACCACAGGCATTGTGATG	TTTGATGTCACGCACGATTTC
Adipoq	GAGAAGGGAGAGAAAGGAGATG	TGAGCGATACACATAAGCGG
Ifng	CTTTGGACCCTCTGACTTGAG	TCAATGACTGTGCCGTGG
Il6	CAAAGCCAGAGTCCTTCAGAG	GTCCTTAGCCACTCCTTCTG
Keap1	CACACTAGAGGATCACACCAA	CCGTGTAGGCGAACTCAATAA
Lep	AGCCTCACTCTACTCCACAG	CTCTACATGATTCTTGGGAGC
Nfe2l2	CTCCGTGGAGTCTTCCATTTAC	GGGCGGCGACTTTATTCTTA
Nox1	TCCTTCGCTTTTATCGCTCC	TCGCTTCCTCATCTGCAATTC
Nox2	TTGTACGTGGACAGACTGCG	GGGTTTCCAGCGAACTTTGG
Nox3	TGCCCTGTACCTCAATTTTCTG	ACACGCATACAAGACCACAG
Nox4	TCCAAGCTCATTTCCCACAG	CGGAGTTCCATTACATCAGAGG
Nr0b2	TCTCTTCTTCCGCCCTATCA	GAGGGATTCTGGCTTGAAAGTA
Nr1h5	TGGATGGTGTAGCAGCTAATG	AAGGCAGAGGAAGGGAAATG
Pparg	TGTTATGGGTGAAACTCTGGG	AGAGCTGATTCCGAAGTTGG
Tbp	AAGAAAGGGAGAATCATGGACC	GAGTAAGTCCTGTGCCGTAAG
Tnf	CTTCTGTCTACTGAACTTCGGG	CAGGCTTGTCACTCGAATTTTG
Rn18S	GAGACTCTGGCATGCTAACTAG	GGACATCTAAGGGCATCACAG
